# Shifts in ecological strategy spectra of typical forest vegetation types across four climatic zones

**DOI:** 10.1038/s41598-021-93722-7

**Published:** 2021-07-08

**Authors:** Xin Han, Jihong Huang, Runguo Zang

**Affiliations:** 1 Key Laboratory of Forest Ecology and Environment of National Forestry and Grassland Administration, Research Institute of Forest Ecology, Environment and Protection, Chinese Academy of Forestry, Beijing, 100091 China; 2 Co-Innovation Center for Sustainable Forestry in Southern China, Nanjing Forestry University, Nanjing, 210037 Jiangsu China

**Keywords:** Ecology, Biodiversity, Biogeography

## Abstract

Ecological strategy spectrum is the relative proportion of species in different categories of ecological strategies in a biotic community. Here, we explored ecological strategy spectra in typical forest vegetation types across four climatic zones in China. We classified ecological strategy categories by using the “StrateFy” ordination method based on three leaf functional traits. Results showed that the predominant ecological strategies of species in the tropical rainforest were CS-selected, and the predominant categories in the evergreen-deciduous broadleaved mixed forest and warm-temperate coniferous-broadleaved mixed forest were CSR and S/CSR categories respectively, whereas those in the cold-temperate coniferous forest were the S-selected ones. Ecological strategy richness of forest vegetation decreased significantly with the increase of latitude. The categories of ecological strategies with more component S increased while those with more component C decreased with the change of typical forest vegetation types from tropical rainforest through evergreen-deciduous broadleaved mixed forest and warm-temperate coniferous-broadleaved mixed forest to cool-temperate coniferous forest. Our findings highlight the usefulness of Grime’s C-S-R scheme for predicting the responses of vegetation to environmental changes, and the results are helpful in further elucidating species coexistence and community assembly in varied climatic and geographic settings.

## Introduction

How plant traits vary among communities has been a major challenge in ecology^1,2^. A better understanding of trait-environment relationships may enhance our ability to predict vegetation responses to both natural and human-generated environmental changes^3^. An ecological strategy can be defined as a group of plant species sharing similar functional traits, similar responses to environmental factors and/or similar roles in an ecosystem or a biome^4–7^. Different ecological strategies represent contrasting approaches of species surviving in varying habitats^8^. Ecological strategy spectrum is defined as the relative proportion of species in different types of ecological strategies in a biotic community and can act as a linkage between functional traits and ecosystem processes, which can be a promising way forward for answering important ecological questions at the level of ecosystems, landscapes or biomes. For example, responses of vegetation to environmental changes (e.g. changes in climate, land use and natural disturbance regimes)^9–11^. Therefore, comparing the functional compositions of vegetation types across different climatic zones, is an effective way in further understanding interactions between ecological strategies of species assemblages and environmental conditions^12,13^.

Understanding the adaptive ecological strategies of vegetation in coping with changing environmental conditions is one of the prominent focuses in ecology^14–16^. The study of ecological strategies originated from a series of theoretical explorations in ecological communities: e.g. life-form spectrum^17^, r/K selection theory^18^ and competitor, stress-tolerator, ruderal (C-S-R) triangle theory (i.e. C-S-R theory)^5,19,20^. The life-form spectrum theory examined variations of plants in different forms of adaptation to the dominant stressful abiotic conditions in vegetation types under varied climatic zones^17^. The r/K selection theory suggest that there are two filtering forces in natural communities, which select for the colonizing and competitive abilities of species^18^. A species with strong colonizing ability may have a large geographical range but have a low dominance in local communities, whereas a species with strong competitive ability may dominate in the local communities, but have a weak colonizing ability across large ranges^21^. On basis of the r/K selection theory, Grime^19,20^ further developed a C-S-R theory for plants^21^ by distinguishing the three major processes that affect plant distributions, i.e. plant responses to, biotic competition, stress tolerance and disturbance regimes. Specifically, C-selected ‘competitors’ are likely to survive in relatively stable and favorable site conditions through investing their resources in vegetative growth and rapid development of large individual size to have a resource preemption. S-selected ‘stress-tolerators’ survive the variable and resource-poor environment conditions by investing their resources mainly in the capacity to obtain more available resources in harsh abiotic conditions. They may be small or gradually attain large size over a long life span. R-selected species, or ruderalism, invest a large proportion of their resources not in the whole body but in their different propagules from which they can regenerate in conditions of repeated, often destructive disturbances^22^. A trait-based analysis of plant adaptations may provide a better insight into community dynamics, because easily measurable functional traits not only co-vary with environmental gradients but also directly reflect the adaptive strategies of different species under varied living conditions^23,24^. Recently, functional traits have been used for identifying ecological strategies, reflecting how species cope with environmental factors^15,20,22,25,26^. Furthermore, global investigation of key vascular plant traits has confirmed that plant functional variations are closely linked with their ecological strategies in adapting changing environmental conditions^27^. Compared to other two ecological strategy theories, C-S-R theory is now widely used to explain and predict processes observed in natural communities^28^.

The C, S and R ecological strategies respectively represent three extremes of evolutionary specialization corresponding to the three extreme habitats which only occupy part of the spectrum of habitats available to plants. Therefore, in addition to the three primary strategies, there are various strategies corresponding to the various habitat conditions which are possible between different combinations of competition, stress, and disturbance. Grime^29^ proposed the ordination of different species of a community in a ternary plot (i.e. C-S-R triangle), which can well reflect the trade-offs of species among tolerance of competition, stress and disturbance. Up to now, several methods for CSR analysis have been developed^30–33^ to assign species to a position in an ordination of C-S-R triangle. However, a wider application and generalization of these methods on basis of the C-S-R theory has some limitations^32,33^, since the traits used were often specific to certain plant organs or plant groups, or the species in particular regions. To overcome these limitations, a CSR-classification method in which a few easily determined leaf traits are compared against the global leaf economics spectrum was developed^33^. Specifically, extremely high values of specific leaf area (SLA) and leaf dry matter content (LDMC) are highly representative of extreme species with fast or slow leaf economics, respectively. Leaf area (LA) determines the ability of species to intercept light, which correlates with plant and seed size, orthogonal to the leaf economics spectrum^27,33,34^. This approach therefore was considered to represent the most prominent aspects of plant functional variation globally^27^. Additionally, previous studies have confirmed that this method is generally applicable to vascular plants, sufficiently precise to distinguish strategies among species within genera, among populations within species and across biomes, and its validity has been confirmed in several experimental and field studies^7,16,35^.

Using the ordination tool^22^, species can be classified into 19 tertiary CSR strategy categories^32^ according to continuous C-, S- and R-scores which are calculated as the percentage of the main strategy types based on the trade-off between the three leaf traits (LA, LDMC and SLA) of plant species. Depending on the readily available traits, the C-S-R classification method (i.e. “StrateFy”) makes it possible to investigate primary ecological strategies across a wide range of species and habitats. C-S-R theory has previously been applied to explore the difference in ecological strategies between forest types, with findings that forests under severe conditions were strongly associated with the S strategy, in contrast to forests under favorable conditions presenting a C strategy, while R strategy was under-represented amongst plant species^22,36^. These results suggest that C-S-R theory can bridge the gap between plant traits and ecosystem processes, providing a powerful tool for studying the interactions of ecological strategies and environmental conditions. Although previous studies addressing the role of ecological strategies compared the proportions of species in each CSR category^37–40^, no study has considered how the ecological strategies of species in different communities based on the 19 categories of tertiary strategies classification changed across biomes under varied climatic conditions. To evaluate the differences in ecological strategies among communities, by analogy with life-form spectrum, we proposed the ecological strategy spectrum based on C-S-R theory, which is the relative richness of species in different types of ecological strategies in a biotic community.

Ecological strategy spectrum of forest vegetation is the result of long-term adaptation of communities to their living environment. Evaluation of ecological strategy spectrum can help to understand plant adaptations to the changing environment. Here, on basis of measuring species trait data from 200 established forest dynamics plots (FDPs) in typical forest vegetation types across four climatic zones (tropical, subtropical, warm-temperate, and cold-temperate), we classified ecological strategies of woody species in different vegetation types across four climatic zones to explore the differentiation pattern of ecological strategy spectra for plant communities along the latitudinal climatic gradient. We hypothesize that the ecological strategy spectrum of typical forest vegetation differentiated significantly across the climatic zones, and categories of ecological strategies with more component S will increase while those with more component C will decrease with the change of typical forest vegetation types from tropical through subtropical and warm-temperate to cool-temperate climatic zones. Our study will provide empirical evidences in predicting vegetation responses to future climate and other environmental changes.

## Material and methods

### Study site

The study was conducted in four typical forest vegetation types across four climatic zones (tropical, subtropical, warm-temperate and cool-temperate) in China. The typical vegetation types were: (A) a tropical rainforest located in Bawangling Nature Reserve in Hainan; (B) a subtropical evergreen-deciduous broadleaved mixed forest located in Mulingzi and Xingdoushan Nature Reserve in Hubei; (C) a warm-temperate coniferous-broadleaved mixed forest located in Xiaolongshan Nature Reserve in Gansu; and (D) a cold- temperate coniferous forest located in Kanasi Nature Reserve in Xinjiang. The four study sites were distributed along a latitudinal gradient from 18°52′N to 49°11′N, and covered the longitudinal range from 86°54′ to 110°17′E across the four climatic zones. The mean annual temperature decreased along the latitudinal gradient from 23.6 to − 0.2 °C. The mean annual precipitation ranged from 800 to 1750 mm. Details of the study sites are shown in Table 1.

**Table 1:** The geographic profiles of typical forest vegetation types across four climatic zones in China.

Climatic zone	Typical forest vegetation type	Location			Climate	
		Nature reserve	Latitude (N)	Longitude (E)	MAT (°C)	MAP (mm)
Tropical	Tropical rainforest	Bawangling	18°52′–19°12′	108°53′–109°20′	23.6	1750
Subtropical	Subtropical evergreen-deciduous broadleaved mixed forest	Mulingzi and Xingdoushan	29°55′–30°10′	108°57′–110°17′	15.5	1733
Warm-temperate	Warm-temperate coniferous-broadleaved mixed forest	Xiaolongshan	33°30′–34°49′	104°22′–105°43′	10.9	800
Cold-temperate	Cold-temperate coniferous forest	Kanasi	48°35′–49°11′	86°54′–87°54′	− 0.2	1065

*MAT* mean annual temperature, *MAP* mean annual precipitation.

### Forest dynamics plots design, species identification and nomenclature

We did our field sampling work in four typical old growth forest vegetation types across four climatic zones (tropical, subtropical, warm-temperate and cool-temperate) in China. When we established our forest dynamics plots in each type of the old growth forests across the four climatic zones, we tried to avoid falling into pseudo-replication in designing the spatial distribution of the plots. We randomly sampled and established fifty 20 m × 20 m permanent forest dynamics plots (FDPs) in each old-growth forest type at each of the four study sites according to the standard protocols of the Center for Tropical Forest Science^41^. In each old growth forest type, we ensured that the distance among each of the 20 m × 20 m plot was more than 100 m. Thus, each of the 50 plots in each forest type were randomly distributed and they had no spatial autocorrelation. In addition, we have done the auto-correlation analysis for each 50 plots in each of the four forest types, the results showed that there were no spatial autocorrelations among the 50 plots within each of the same forest type (see the supporting information S1). In each FDP, all woody plants (trees, shrubs, and lianas) with a diameter at breast height (DBH) ≥ 1 cm were tagged, mapped, identified and their DBH were measured.

All woody plant species in our study were identified to species level by our investigation team in the field with the help of local botanists (including the Xiusen Yang in Hainan, Yongmei Yi in Hubei Province, Anmin Li in Gansu). The plant species in Xinjiang were identified to species level by ourselves. All voucher specimens were deposited at the Herbarium of Chinese Academy of Forestry, the Herbarium of South China Botanical Garden, Chinese Academy of Sciences and the Herbarium of Institute of Botany, the Chinese Academy of Sciences. The botanical nomenclature of species in the database was standardized according to the Flora of China (http://www.efloras.org).

### Functional traits

We measured the three leaf functional traits of all woody plants in each FDP: leaf area (LA, mm^2^, i.e. the mean surface area of the one-sided area of a single fully expanded fresh leaf), specific leaf area (SLA, mm^2^ mg^−1^, i.e. LA divided by leaf dry weight (LDW)) and leaf dry mass content (LDMC, %, i.e. LDW divided by leaf fresh weight (LFW)). At least 10 individuals of each species were sampled for measurement of functional traits. For those species with an abundance of less than ten stems, all the individuals were sampled. We selected the fully expanded leaves from adult plants in the field, avoiding leaves with obvious symptoms of pathogen or herbivore attack, or with a substantial cover of epiphylls. We sampled sun leaves from plants growing under relatively optimal conditions. For species that typically grow in the overstory, we took leaves from plant parts most exposed to direct sunlight. To keep the leaf material collected from plants growing in situ fresh, plant material was stored in plastic bags and transported to the laboratory for being measured within 24 h.

We chose two healthy leaves from each individual. We weighted leaf fresh weight (LFW, mg) and then scanned the leaves using a flatbed scanner and calculated LA using ImageJ (from the US National Institutes of Health; http://www.nih.gov/, accessed 22 February 2013). After area measurement, each leaf sample was put in the oven at 80 °C for 48 h; then we determined the leaf dry weight (LDW, mg) and calculated the parameters SLA and LDMC. All of the sampling and measurement methods were following the criteria of Pérez-Harguindeguy^42^. Given that lianas are sampled difficultly leading to functional traits of lianas severe missing, only trees and shrubs were considered in this study.

### Data and statistical analysis

According to the scheme of Hodgson^32^, ecological strategies could be categorized into 19 types, including 3 primary (C, S, and R), 4 secondary (CS, CR, SR and CSR) and 12 tertiary (C/CR, C/CS, C/CSR, CR/CSR, CS/CSR, R/CSR, S/CS, S/CSR, S/SR, SR/CSR, R/CR and R/SR). “StrateFy” as proposed by Pierce^22^ et al. (2017) was used to classify ecological strategies for each species included in our study, which was based on three functional traits LA, LDMC and SLA.

We use the “strategy richness” to reflect the number of strategy categories^32,43^ (i.e. Hodgson’s 19 tertiary CSR strategy categories) presenting in each FDP (e.g. C, S, R, CS, CSR, R/CSR, etc.). The ecological spectrum of each FDP is obtained by calculating the number of species belonging to certain strategy category divided by total number of species present in a community. To detect whether strategy richness and ecological strategy spectrum differ among typical forest vegetation types across different climatic zones, we performed LMM (Linear Mixed Models) using the plots in each forest type as a random effect.

We performed all statistical analyses by using the R 3.6.1 Program^44^. We created ternary graphs by using “ggtern” function of the “ggtern” package^45^. The difference analyses of ecological strategy richness and spectra were conducted by using the “lme” function of the “nlme” package^46^ and “ggbarplot” function of the “ggpubr” package^47^.

### Ethical statement

The collections of leaf material in this research were permitted by four provincial Forestry and Grassland Administrations in China, including Hainan Province, Hubei Province, Gansu Province and Xinjiang Uygur Autonomous Region. And all surveys comply with relevant institutional, national, and international guidelines and legislation. The authors are responsible for all aspects of the work.

## Results

### Distribution of species in the C-S-R triangle in different forest vegetation types across the climatic zones

**Figure 1:**
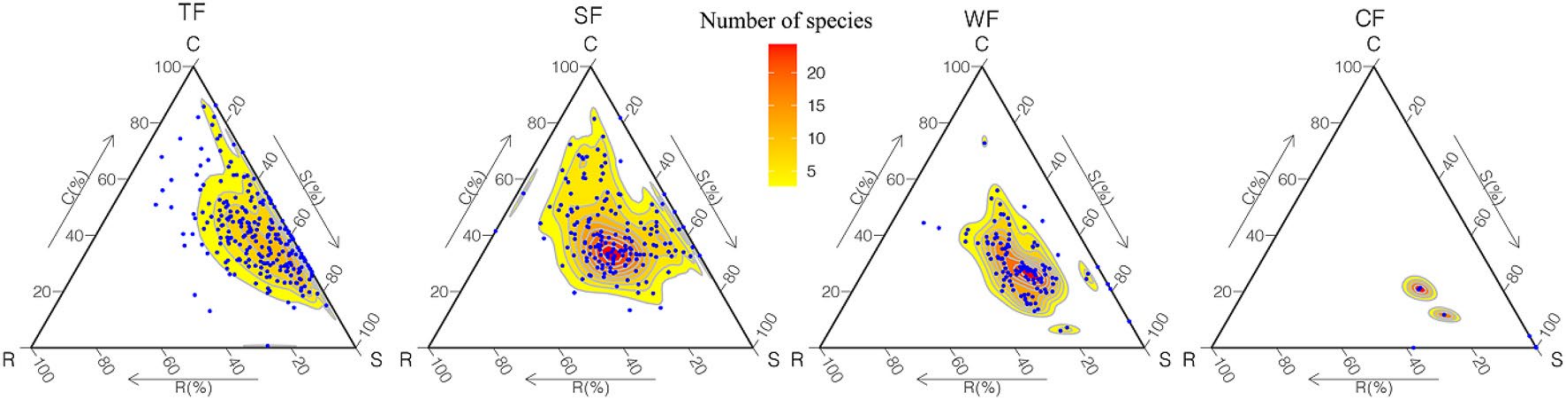
Distribution of species’ ecological strategies in the C-S-R triangle of typical forest vegetation types across four climatic zones in China. *TF* tropical rainforest, *SF* subtropical evergreen-deciduous broadleaved mixed forest, *WF* warm-temperate coniferous-broadleaved mixed forest, *CF* cold-temperate coniferous forest. The numbers of species gradient in typical forest vegetation types are visualized by colored polygon. Blue dot represents individual species. Deeper shades of color correspond with greater number of species. The C (%), S (%) and R (%) each represent the components C, S and R in C-S-R triangle.

In the ternary plots (Fig. 1), there are obvious differences in the distribution of species in different ecological strategy categories of different forest vegetation types across the four climatic zones.

In tropical rainforest, species are mainly clustered in C-S zone in the C-S-R triangle. Species in subtropical evergreen-deciduous broadleaved mixed forest and warm-temperate coniferous-broadleaved mixed forest mainly distributed in the center of C-S-R triangle but had more species distributed toward the C-corner, however, species in warm-temperate coniferous-broadleaved mixed forest had a distribution tendency of more species approaching the S-corner. Distribution of species in cold-temperate coniferous forest was concentrated to the S-corner. However, there was no species tending to distribute toward the R-corner in C-S-R triangle for all the typical forest vegetation types. As the climatic zones changes from tropical to subtropical to warm-temperate to cool-temperate in China, the ecological strategies of species in the typical forest vegetation types shifted gradually from C-S zone through C-S-R zone to S-corner in C-S-R triangle.

### Changes of ecological strategy richness in different forest vegetation types across the climatic zones

**Figure 2:**
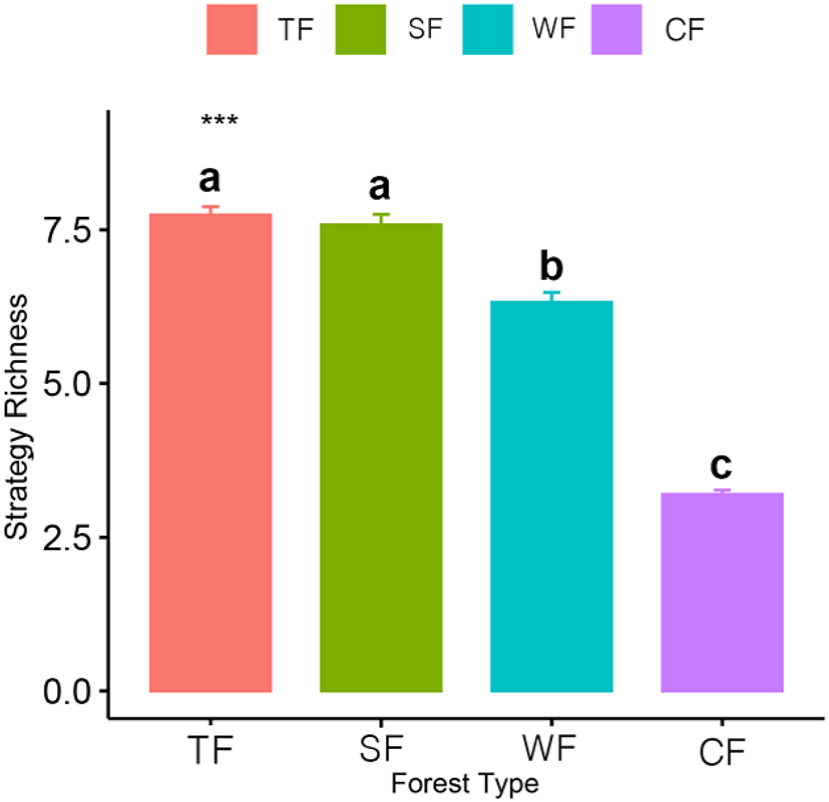
Variations in ecological strategy richness in different forest vegetation types across four climatic zones in China. *TF* tropical rainforest, Red, *SF* subtropical evergreen-deciduous broadleaved mixed forest, Green, *WF* warm-temperate coniferous-broadleaved mixed forest, Blue, *CF* cold-temperate coniferous forest, Purple. Number of replicates N = 50 and means and standard errors are given. *** (P < 0.001) indicates significant difference in ecological strategy richness in different forest vegetation types across four climatic zones in China. Different letters denote significant difference between typical forest vegetation types (P < 0.05).

There were obvious differences in the ecological strategy richness (i.e. the number of strategies) of different forest vegetation types across the four climatic zones (Fig. 2).

Ecological strategy richness differed significantly between the typical forest vegetation types except between tropical rainforest and subtropical evergreen-deciduous broadleaved mixed forest. Furthermore, ecological strategy richness of different forest vegetation types decreased significantly with an increase in latitude.

### Variations of ecological strategy spectrum of different forest vegetation types across the climatic zones

**Figure 3:**
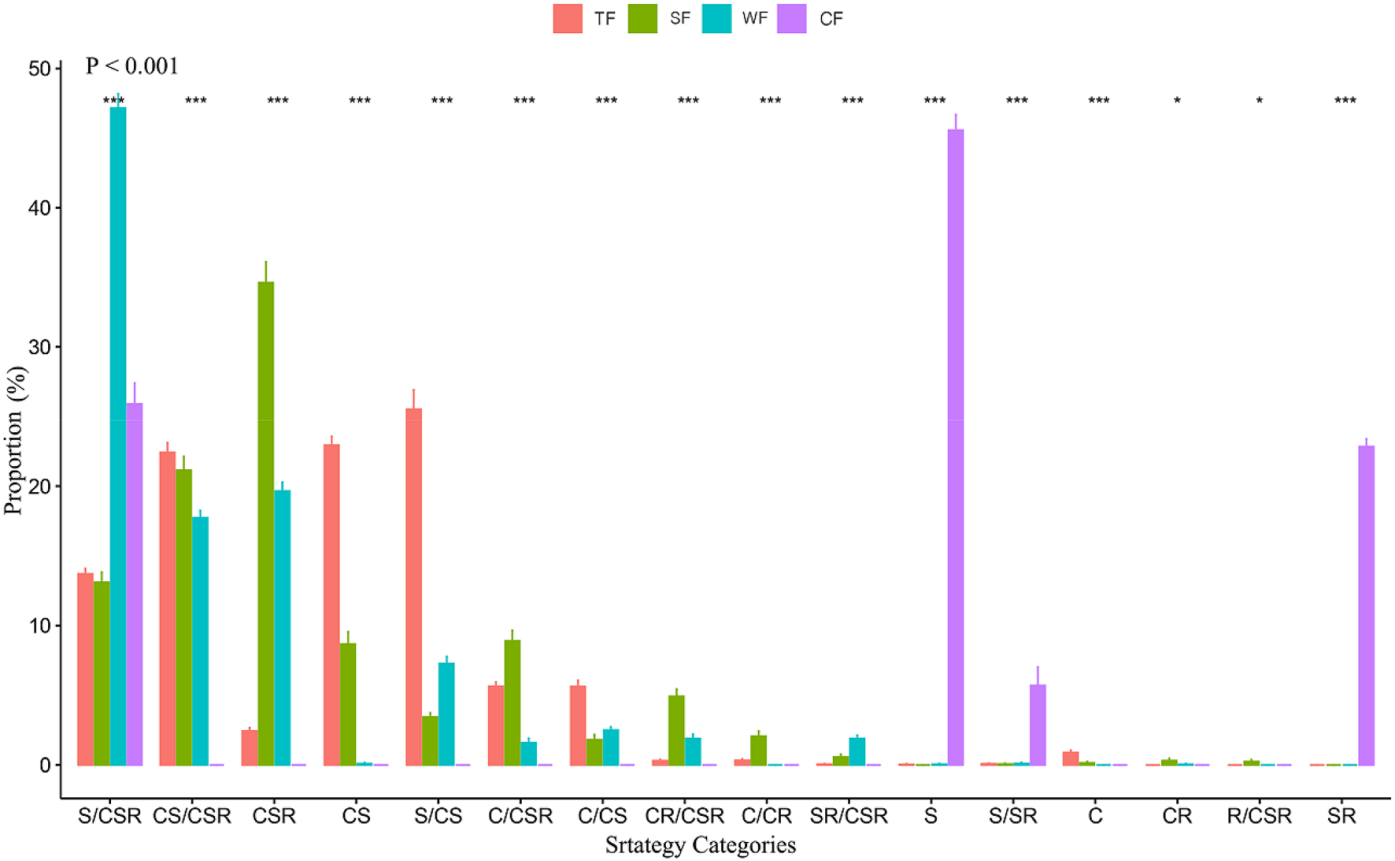
Variations of ecological strategy spectra in different forest vegetation types across four climatic zones in China. *TF* tropical rainforest, Red, *SF* subtropical evergreen-deciduous broadleaved mixed forest, Green, *WF* warm-temperate coniferous-broadleaved mixed forest, Blue, *CF* cold-temperate coniferous forest, Purple. The proportion (%) indicate the average of the relative number of species in each strategy category. Strategy categories indicates different ecological strategy: C indicates competitor, S indicates stress-tolerator and R indicates ruderal and more detail description refer to C-S-R classification after Hodgson^32^. Number of replicates N = 50 and means & standard errors are given. *** (P < 0.001) and * (P < 0.05) indicates significant difference in different strategy category among typical forest vegetation types. P < 0.001 in the top left indicates significant difference among ecological strategy spectra of typical forest vegetation types.

Ecological strategy spectra differed significantly in different forest vegetation types across the four climatic zones (Fig. 3).

CS-selected strategies dominated in ecological strategy spectrum of tropical rainforest, while S-selected strategies dominated in cold-temperature coniferous forest. Specifically, the proportions of CS and S/CS strategy were largest in tropical rainforest, while the proportion of S strategy was largest in cold-temperature coniferous forest (Fig. 3). Unlike the above two extreme typical forest vegetation types, CSR-selected strategies (e.g. CSR and S/CSR) dominated in the ecological strategy spectra of subtropical evergreen-deciduous broadleaved mixed forest and warm-temperate coniferous-broadleaved mixed forest. The proportions of CSR and S/CSR strategy were largest respectively in subtropical evergreen-deciduous broadleaved mixed forest and warm-temperate coniferous-broadleaved mixed forest (Fig. 3).

## Discussion

Overall, we found significant differences in the distribution pattern of species in different ecological strategy categories among the typical forest vegetation types across four climatic zones in China. Specifically, the dominant ecological strategies in the tropical rainforest were CS-selected strategies (e.g. CS and S/CS), and the dominant ecological strategies in the evergreen-deciduous broadleaved mixed forest and warm-temperate coniferous-broadleaved mixed forest were CSR-selected strategies (e.g. CSR and S/CSR), whereas those in cold-temperate coniferous forest were S-selected strategies (e.g. S; Fig. 1). In addition, ecological strategy richness of typical forest vegetation types in China decreased gradually from tropical though subtropical and warm-temperate to cold-temperate climatic zones (Fig. 2). Moreover, significant differences existed in the ecological strategy spectra of different forest vegetation types across the four climatic zones in China (Fig. 3).

### Distribution pattern of species’ ecological strategy in different forest vegetation types across the climatic zones

Consistent with our hypotheses, the typical forest vegetation types across different climatic zones exhibited different distribution pattern of ecological strategies most species in tropical rainforest were classified as CS-selected strategies, CSR-selected strategies were evident in evergreen-deciduous broadleaved mixed forest and warm-temperate coniferous-broadleaved mixed forest, while for cold-temperate coniferous forest, S-selected strategies dominated (Fig. 1). This confirmed previous theoretical and empirical work that certain biomes are characterized by relatively functionally restricted floras (i.e. clustered within particular zones of the C-S-R triangle) based on the analysis to biomes world-wide^22^. Plant functional traits are the foundation for constructing C-S-R classifications to identify ecological strategies^2,29^, all of the involved traits are frequently associated with environmental changes^42,48,49^. Some researches demonstrated that functional traits (e.g. SLA) were directly influenced by climatic factors^50,51^ (e.g. temperature or precipitation). For example, SLA decreases as temperature increases based on the study of more than 2500 vascular plants at the global scale^49^ and LDMC decreases as precipitation increases^52^. Since our study is based on the typical forest vegetation types across four climatic zones, which reflects the climatic shifts from cold, dry to warmer, moister conditions along the north-to-south gradients in China^53^. Moreover, environmental filter leads to convergence in leaf functional traits within biomes and divergence between biomes across global scale^54,55^. Therefore, species in the same forest vegetation types clustered to certain zones in the C-S-R triangle.

In our study, we found that there are very few R-selected strategies occurring in all of the typical forest vegetation types across different climatic zones. This is consistent with previous research^36^ in subtropical forests in southern Brazil. Very few R-selected strategies in forest vegetation types may result from that the woody growth forms in forests exhibited few R strategies^21,22^. For example, trees were clustered around a CS strategy and without R-selected trees apparent, and shrubs centred around an S/CSR strategy, while herbs exhibited an R/CSR strategy^22^. Additionally, R-selected species mostly dominate in pioneer vegetation, while in later stages of succession S-selected species strongly dominate^5^. In this paper, the species involved are woody species and the typical forest vegetation types are in late successional stage, i.e. old growth forests. Therefore, few R-selected species existed in these typical forest vegetation types.

### Changes of ecological strategy richness in different forest vegetation types across the climatic zones

The ecological strategy richness significantly decreased gradually from tropical, through subtropical and warm-temperate to cold-temperate climatic zone (Fig. 2). That is probably because the variations of traits which highly represent ecological strategies occur concurrently within and between different vegetation types^56^. On the one hand, ample evidences indicate that plant traits and trait syndromes are significantly affected by climate changes^27,54,57^. Under natural conditions, different climatic conditions lead to species with similar traits and trait syndromes clustered, shaping the large-scale geographical pattern of vegetation types^53^ and further lead to ecological strategies variation among different forest vegetation types. On the other hand, according to Grime’s view^58^, temporal and spatial heterogeneity of ecosystems allows some species with unique traits to germinate and survive in specific micro-niches^59^. Moreover, different from environmental filtering between biomes, within community environmental heterogeneity could potentially promote the strong trait divergence^56^. For instance, compared with cold-temperate coniferous forest, the more heterogeneity of light intensity in canopy, understorey and gap in tropical rainforest leads to trait divergence in the regional pool of species^56,60^ and therefore more ecological strategy richness possibly occurring in tropical rainforest than in cold-temperate coniferous forest. These suggest that the combined effects of macro-heterogeneity (i.e. between forest vegetation types across different climatic zones) and micro-heterogeneity (i.e. within community) can provide more survival opportunities for diverse species with various traits and thus make more diverse ecological strategies emerge. Besides, Cerabolini^43^ found a significant positive correlation between ecological strategy richness and species richness. In this study, the species richness of typical forest vegetation types gradually decreases along the latitudinal gradients, which may also contribute to a gradual decrease in ecological strategy richness of the typical forest vegetation types across the climatic zones in China.

### Variations of ecological strategy spectrum in different forest vegetation types across the climatic zones

To our knowledge, no study has explicitly examined the differences in ecological strategy spectra of different forest vegetation types based on the C-S-R theory across different climatic zones. Our results indicate that significant differences exist in ecological strategy spectra among typical forest vegetation types across different climatic zones in China. These significant differences among typical forest vegetation types across different climatic zones can be explained as follows. On the one hand, according to the above findings that both macro-heterogeneity (i.e. across climatic zones) and micro-heterogeneity (within forest communities), abiotic filtering played important roles in the variations of functional traits^51,54,61^, which lead to differences in distribution pattern of ecological strategies and ecological strategy richness (Figs. 1 and 2). These in turn contributed to a significant part of differences in ecological strategy spectra among different forest vegetation types across the climatic zones in China.

On the other hand, we considered that different adaption of ecological strategies of species to varied environmental conditions also might influence the ecological strategy spectra among different forest vegetation types across different climatic zones. The C-S-R theory^2,5,19,20^ suggested that ecological strategies of species could reflect species abilities to cope with the environmental changes. The primary strategies (C, S, and R) are assumed to adapt to conditions experiencing extreme intensities of competition, stress or disturbance. Secondary strategies (CS, CR, SR and CSR) are adapted to conditions experiencing differing combinations of competition, stress and disturbance. Specifically, CS adapted to relatively-undisturbed conditions experiencing moderate intensities of stress; CSR strategy adapted to conditions in which the level of competition is restricted by moderate intensities of both stress and disturbance. In this study, for all typical forest vegetation types are old-growth without any apparent anthropogenic disturbances, competition and stress maybe more important process in explaining the variations of ecological strategy spectra among typical forest vegetation types. Tropical rainforest in tropical climatic zones, with high temperatures and moisture^36^ (Rosenfield et al. 2019) but variable soil fertility^62^ (Ding et al. 2017), favored CS strategy which is simultaneously with high competition and stress tolerance to survive and grow^22^. By contrast, cold-temperate coniferous forest in cold-temperate climatic zones, with low temperature and water deprivations^24^, favored species with extremely high stress tolerance (e.g. S strategy) to cluster^5,27^. Therefore, CS strategy dominated in tropical rainforest, whereas S strategy dominated in cold-temperate coniferous forest in our study.

Unlike the above two forest types, subtropical evergreen-deciduous broadleaved mixed forest and warm-temperate coniferous-broadleaved mixed forest are respectively in subtropical and warm-temperate climatic zones. Both of them are located at an intermediate position along the latitudinal and environmental gradients. Abiotic and biotic conditions are relatively favorable and high availability of resources compared with tropical rainforest and cold-temperate coniferous forest^24^, leading to that species in these two forest types do not need to be highly competitive or stress tolerant to survive and grow. Meanwhile, probably due to the strong trade-off among the C, S and R strategies, CSR-selected strategies (e.g. CSR and S/CSR) dominated in the two forest types. In addition, deciduous tree species usually adopt ecological strategies with high growth rates^20^, while coniferous species usually maintain relatively high stress tolerance^11^. This might partly explain that the proportion of CSR strategy and S/CSR strategy are largest in subtropical evergreen-deciduous broadleaved mixed forest and warm-temperate coniferous-broadleaved mixed forest, respectively.

In conclusion, our study shows that the ecological strategy richness and ecological strategy spectra, assessed by Grime’s C-S-R theory, are significantly different among the typical forest vegetation types across different climatic zones in China. Our findings highlight the usefulness of Grime’s C-S-R theory for predicting the responses of the forest vegetation to environmental changes, as ecological strategies in different species assemblages depend on the interactions among multiple traits and varied environmental variables^36^. Our results are helpful for ecologists to further understanding mechanism of species coexistence and community assembly in a changing climate, such as predicting which kinds of species may assemble in certain biogeography in response to changes in climatic conditions.

## Supplementary Information

Supplementary Information.
