# Supplementary material for: Shifts in ecological strategy spectra of typical forest vegetation types across four climatic zones
Source: Sci Rep. 2021 Jul 8;11:14127. doi: 10.1038/s41598-021-93722-7 (PMC8266834; doi:10.1038/s41598-021-93722-7)

Supplementary Figure 1: Mantel Correlogram for spatial autocorrelation analysis. Mantel correlation between dissimilarity in ecological strategy spectra and distance among plots is shown for each distance class. Blank squares indicate that there were no significant spatial autocorrelations among the 50 plots within each of the same forest type. (TF, tropical rainforest; SF, subtropical evergreen-deciduous broadleaved mixed forest; WF, warm-temperate coniferous-broadleaved mixed forest; CF, cold-temperate coniferous forest)

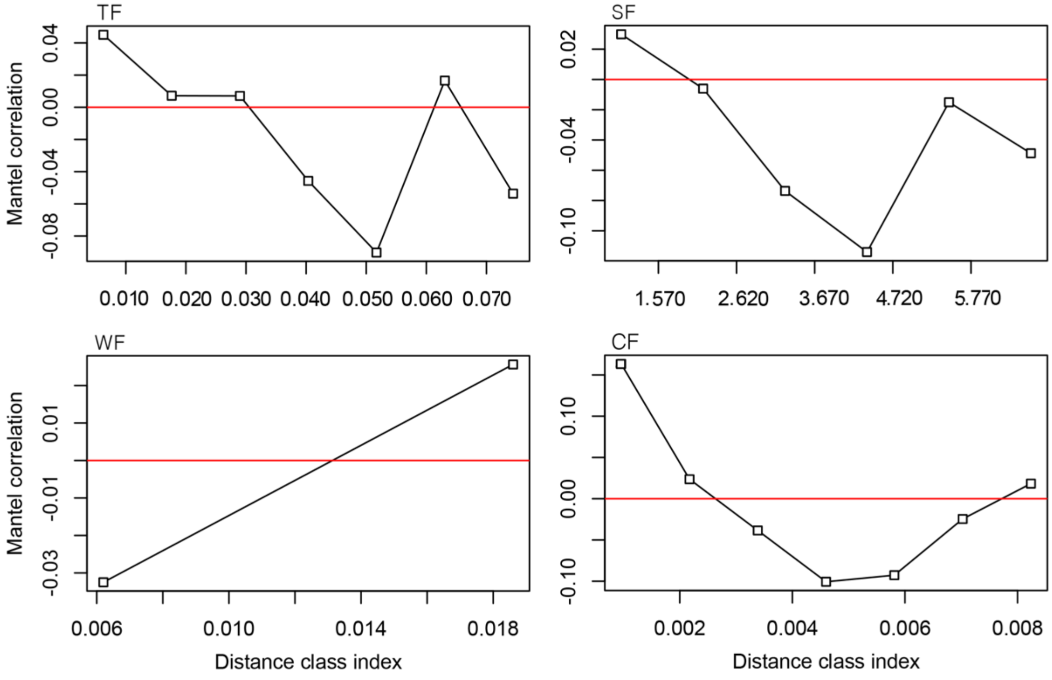

Supplement: Supplementary file 1 — Supplementary Information. [file 41598_2021_93722_MOESM1_ESM.pdf]
